# Use of General Primary Care, Specialized Primary Care, and Other Veterans Affairs Services Among High-Risk Veterans

**DOI:** 10.1001/jamanetworkopen.2020.8120

**Published:** 2020-06-29

**Authors:** Evelyn T. Chang, Donna M. Zulman, Karin M. Nelson, Ann-Marie Rosland, David A. Ganz, Stephan D. Fihn, Rebecca Piegari, Lisa V. Rubenstein

**Affiliations:** 1 Center for the Study of Healthcare Innovation, Implementation and Policy, Veterans Affairs (VA) Greater Los Angeles Healthcare System, Los Angeles, California; 2 Division of General Internal Medicine, VA Greater Los Angeles Healthcare System, Los Angeles, California; 3 Division of General Internal Medicine, David Geffen School of Medicine at UCLA (University of California at Los Angeles), Los Angeles; 4 Center for Innovation to Implementation, VA Palo Alto Health Care System, Menlo Park, California; 5 Division of Primary Care and Population Health, Stanford University School of Medicine, Stanford, California; 6 Seattle-Denver Health Services Research & Development Center of Innovation, VA Puget Sound Healthcare System, Seattle, Washington; 7 General Internal Medicine Service, VA Puget Sound Healthcare System, Seattle, Washington; 8 Department of Medicine, University of Washington, Seattle; 9 Department of Health Services, University of Washington, Seattle; 10 VA Pittsburgh Center for Health Equity Research and Promotion, Pittsburgh, Pennsylvania; 11 Department of Medicine, School of Medicine, University of Pittsburgh, Pittsburgh, Pennsylvania; 12 VA Greater Los Angeles Geriatric Research, Education and Clinical Center, Los Angeles, California; 13 UCLA Multicampus Program in Geriatric Medicine and Gerontology, Los Angeles, California; 14 VA Office of Clinical Systems Development & Evaluation, Washington, DC; 15 Fielding School of Public Health, UCLA, Los Angeles, California; 16 RAND Corporation, Santa Monica, California

## Abstract

**Importance:**

Integrated health care systems increasingly focus on improving outcomes among patients at high risk for hospitalization. Examining patterns of where patients obtain care could give health care systems insight into how to develop approaches for high-risk patient care; however, such information is rarely described.

**Objective:**

To assess use of general and specialized primary care, medical specialty, and mental health services among patients at high risk of hospitalization in the Veterans Health Administration (VHA).

**Design, Setting, and Participants:**

This national, population-based, retrospective cross-sectional study included all veterans enrolled in any type of VHA primary care service as of September 30, 2015. Data analysis was performed from April 1, 2016, to January 1, 2019.

**Exposures:**

Risk of hospitalization and assignment to general vs specialized primary care.

**Main Outcome and Measures:**

High-risk veterans were defined as those who had the 5% highest risk of near-term hospitalization based on a validated risk prediction model; all others were considered low risk. Health care service use was measured by the number of encounters in general primary care, specialized primary care, medical specialty, mental health, emergency department, and add-on intensive management services (eg, telehealth and palliative care).

**Results:**

The study assessed 4 309 192 veterans (mean [SD] age, 62.6 [16.0] years; 93% male). Male veterans (93%; odds ratio [OR], 1.11; 95% CI, 1.10-1.13), unmarried veterans (63%; OR, 2.30; 95% CI, 2.32-2.35), those older than 45 years (94%; 45-65 years of age: OR, 3.49 [95% CI, 3.44-3.54]; 66-75 years of age: OR, 3.04 [95% CI, 3.00-3.09]; and >75 years of age: OR, 2.42 [95% CI, 2.38-2.46]), black veterans (23%; OR, 1.63; 95% CI, 1.61-1.64), and those with medical comorbidities (asthma or chronic obstructive pulmonary disease: 33%; OR, 4.03 [95% CI, 4.00-4.06]; schizophrenia: 4%; OR, 5.14 [95% CI, 5.05-5.22]; depression: 42%; OR, 3.10 [95% CI, 3.08-3.13]; and alcohol abuse: 20%; OR, 4.54 [95% CI, 4.50-4.59]) were more likely to be high risk (n = 351 012). Most (308 433 [88%]) high-risk veterans were assigned to general primary care; the remaining 12% (42 579 of 363 561) were assigned to specialized primary care (eg, women’s health and homelessness). High-risk patients assigned to general primary care had more frequent primary care visits (mean [SD], 6.9 [6.5] per year) than those assigned to specialized primary care (mean [SD], 6.3 [7.3] per year; *P* < .001). They also had more medical specialty care visits (mean [SD], 4.4 [5.9] vs 3.7 [5.4] per year; *P* < .001) and fewer mental health visits (mean [SD], 9.0 [21.6] vs 11.3 [23.9] per year; *P* < .001). Use of intensive supplementary outpatient services was low overall.

**Conclusions and Relevance:**

The findings suggest that, in integrated health care systems, approaches to support high-risk patient care should be embedded within general primary care and mental health care if they are to improve outcomes for high-risk patient populations.

## Key Points

**Question:**

Within the Veterans Health Administration, what is the role of general primary care, specialized primary care, mental health, and medical specialty services in caring for veterans at high risk for hospitalization?

**Findings:**

In this cross-sectional study, veterans at high risk for hospitalization had significantly more mental health encounters than primary care encounters and significantly more primary care encounters than medical specialty encounters. Most high-risk veterans (88%) were cared for in general primary care rather than in specialized primary care.

**Meaning:**

The findings suggest that health care system leaders should recognize the critical roles of general primary care and mental health for high-risk patients.

## Teaser

This cross-sectional study assesses the use of general and specialized primary care, medical specialty, and mental health services among patients aat high risk of hospitalization in the Veterans Health Administration.

## Introduction

Patients at high risk for hospitalization are heterogeneous^1^ and complex to treat,^2,3^ and they typically have multiple chronic medical conditions, often compounded by mental health conditions.^4,5,6^ A frequent assumption has been that many of these high-risk patients receive mostly acute emergency care.^7^ Another common assumption has been that high-risk patients with complex conditions receive care mostly through medical specialists,^8,9^ whereas primary care is predominantly directed at a lower-risk population. High-risk patients are often assumed to have low levels of health literacy and to be uninterested in electronic communication with their health care practitioners.^10^ We aimed to inform health care system level planning for high-risk patient care by testing assumptions about whether, where, and how the more than 350 000 highest-risk patients cared for nationally in the Veterans Health Administration (VHA) receive outpatient care.

Primary care settings in integrated health care systems aim to provide care that is coordinated, comprehensive, continuous, and assessible.^11^ Specialty or subspecialty settings, on the other hand, predominantly aim to provide accessible, short-term consultative care. Even when specialists provide long-term continuity care, as they often do for some patients,^8,12,13,14^ they typically do not aim to provide full primary care, defined by the Institute of Medicine as “the provision of integrated, accessible health care services by clinicians who are accountable for addressing a large majority of personal health care needs.”^15^^(p1)^^16^ Sometimes, however, specialists or specialized teams serve as alternatives to general primary care. These specialized primary care settings aim to provide comprehensive, continuous primary care to a population with special needs, such as those with HIV infection,^17^ homeless veterans,^18^ or those with serious mental illnesses.^19^

In this study, we sought to characterize patterns of care for the top 5% of the highest-risk patients enrolled in the VHA nationally based on a predictive risk score for near-term hospitalization that is generated for all VHA patients. We expected that high-risk patients, compared with low-risk patients, would use more face-to-face and fewer secure message encounters with primary care. We also expected that half of high-risk patients would be cared for in specialized primary care settings and that more than half of specialized primary care patients would be high risk. We hypothesized that high-risk patients assigned to general primary care, compared with those assigned to specialized primary care, would have both more primary care and more medical specialty care visits. We aimed to compare (1) primary care encounters (face-to-face, telephone, and secure messages) among high-risk vs low-risk patients, (2) the proportions of high-risk patients assigned to general vs specialized primary care, and (3) the use of primary care and medical (nonsurgical) specialty care visits among high-risk patients assigned to general vs specialized primary care.

## Methods

### Patients 

For this cross-sectional study, we used national electronic administrative data from the VHA Corporate Data Warehouse for veterans enrolled in the VHA in 2015. The study population included all VHA patients assigned to primary care (general or specialized) as of September 30, 2015 (n = 4 309 192). This evaluation was designed to support VHA operations under the authority of VHA National Office of Primary Care.^20^ Patient consent was waived with permission of the VHA National Office of Primary Care because this study was designed for internal VA purposes in support of the VA mission. The findings were designed to be used by and within VA for program and planning purposes. Data were deidentified. This study followed the Strengthening the Reporting of Observational Studies in Epidemiology (STROBE) reporting guideline.

### Setting

Since national implementation of the patient-centered medical home model in more than 900 locations in the VHA in 2010,^21,22^ nearly all patients have been assigned at enrollment to a continuity primary care practitioner (physician, nurse practitioner, or physician assistant) who works with a care team that includes a registered nurse care manager, licensed practical nurse, and clerk.^19,21,23,24,25,26,27^ Patients are assigned to a general or specialized primary care team and may switch to another general or specialized primary care team at any time after enrollment. All teams are tasked with providing comprehensive primary care, including screening and preventive care services.^27,28^ Most general primary care sites are community based, whereas most specialized primary care sites other than those for women’s health are medical center based.^29^

As an integrated health care system, the VHA provides a full range of specialty services, including consultative medical and surgical specialty care as well as integrated primary care and mental health care.^30^ In addition, the VHA offers supplementary services for intensifying care, such as telehealth (remote monitoring for chronic medical conditions),^31^ palliative care, and housing services. The VHA offers multiple modalities to access primary care, including telephone and secure messaging.

### Measures

#### Exposures

We assessed assignment to the type of primary care setting on September 30, 2015, based on administrative data. We assessed for assignment to general primary care and 7 diverse but commonly available types of specialized primary care that together accounted for 98% of patients enrolled in any type of VHA primary care.^32^ We focused on geriatric, homelessness, and women’s health settings as cognitive, nonprocedural services; HIV and dialysis as technical, specialized services; and home-based care and spinal cord injury as disability-focused services.

#### Risk Level

The VHA calculates Care Assessment Needs (CAN) scores^28,33^ weekly for all enrolled veterans who are assigned to any (general or specialized) primary care practitioner and are not hospitalized at the time of CAN score generation. We used an updated CAN model^33^ (eTable 1 in the Supplement) to assess hospitalization risk using VHA administrative data on demographics, use of VHA health services, comorbidity indicators, prescribed medications, vital signs, and veteran-specific variables. The top 5% of patients identified by CAN score have an almost 20% risk of VHA hospitalization within the following 90 days.^28^

We defined the high-risk patient cohort as all patients with a CAN score in the 95th or greater percentile at any time during April 1, 2015, through September 30, 2015 (n = 351 012). We excluded patients who had died during this period for the purpose of assigning a CAN score. We defined the remainder of patients (n = 3 958 180) as low risk. We used the first date that a patient qualified as high risk and the last date (ie, September 30, 2015) for low-risk patients as the index dates for our analyses.

#### Patient Characteristics

We assessed sample patient demographics based on administrative data and medical and psychiatric comorbidities using *International Classification of Diseases, Ninth Revision (ICD-9)* codes (eTable 2 in the Supplement) during the year before the patient’s index date. Housing stability was defined by a combination of *ICD-9* code or receipt of housing services in outpatient encounter data.^34^ We assessed potentially qualifying characteristics for 4 types of specialized primary care (geriatrics, homelessness, women’s health, and HIV) based on age (>70 years of age), housing instability (defined above), sex (female), and HIV status (defined by *ICD-9* code).

#### Use of VHA Services

We counted all patient encounters coded during 2 periods: 1 year before each patient’s index date for high-risk identification (in 2015) and 1 year after the VHA fiscal year 2016 (October 1, 2015, to September 30, 2016). We used the VA Support Service Center^35^ data definitions for primary care encounter types (in-person or telephone) as recorded in the VHA Corporate Data Warehouse.^36^ We used the My HealtheVet patient portal database for data related to secure messages. We counted only 1 encounter per encounter type for any single day. We also counted all nonsurgical specialty encounters (including mental health), inpatient admissions, and emergency department encounters. We performed a sensitivity analysis on use among patients who were alive throughout the VHA fiscal year 2016 (99.4% of the high-risk cohort) and found minor changes (eTable 4 and eTable 5 in the Supplement).

#### Outpatient Encounters

We categorized outpatient encounters into the following 5 mutually exclusive and collectively exhaustive groups using established data definitions during VHA fiscal year 2016^37^: any in-person primary care (general or specialized), any in-person mental health (primary care and mental health integration,^30^ individual and group psychotherapy, or substance use), any in-person medical specialty (excluding encounters related to procedures and chemotherapy), emergency department, or all other (eg, any telephone, surgical, physical therapy, occupational therapy, dental, nutrition, anticoagulation clinic, procedures, chemotherapy, telehealth, and radiology).

#### Add-on Services

We defined add-on services as those designed to supplement primary care for added care intensity. We assessed use of 4 add-on services during VHA fiscal year 2016 with particular relevance to high-risk patients: palliative care, telehealth disease management for chronic conditions, intensive mental health case management (similar to assertive community treatment),^38^ and housing services.

### Statistical Analysis

We used unadjusted logistic regression to generate odds ratios (ORs) for being a high-risk patient (defined by the CAN score) based on veteran characteristics (eg, sex, age, race/ethnicity, and medical condition). We used 2-sample *t* tests to assess differences between veterans at high risk for hospitalization vs those at low risk for hospitalization in rates of primary care visits, emergency department visits, and in-patient discharges. We tested mean use differences between patients assigned to general vs specialized primary care using 2-sample *t* tests and 1-way analysis of variance using the Tukey multiple comparison test.^39^ For all statistical tests, we used a 2-sided *P* < .05 as the a priori significance level and performed 2-tailed hypothesis tests. We performed successive analyses from April 1, 2016, to January 1, 2019. Analyses were conducted with SAS software, version 9.4 (SAS Institute Inc).

## Results

 The study assessed 4 309 192 veterans (mean [SD] age, 62.6 [16] years; 93% male). Male veterans (93%; odds ratio [OR], 1.11; 95% CI, 1.10-1.13), unmarried veterans (63%; OR, 2.30; 95% CI, 2.32-2.35), those older than 45 years (94%; 45-65 years of age: OR, 3.49 [95% CI, 3.44-3.54]; 66-75 years of age: OR, 3.04 [95% CI, 3.00-3.09]; and >75 years of age: OR, 2.42 [95% CI, 2.38-2.46]), black veterans (23%; OR, 1.63; 95% CI, 1.61-1.64), and those with medical comorbidities (asthma or chronic obstructive pulmonary disease: 33%; OR, 4.03 [95% CI, 4.00-4.06]; schizophrenia: 4%; OR, 5.14 [95% CI, 5.05-5.22]; depression: 42%; OR, 3.10 [95% CI, 3.08-3.13]; and alcohol abuse: 20%; OR, 4.54 [95% CI, 4.50-4.59]) were more likely to be high risk (n = 351 012) (Table 1).Compared with low-risk patients (n = 3 958 180), high-risk patients (n = 351 012) had 2.5 times the face-to-face, 4 times the telephone, and twice the number of secure messaging encounters in primary care during the year before being identified as being high risk for hospitalization. Based on the CAN score, which includes rates of acute care use,^33^ high-risk patients had substantially more mean hospital visits (0.8 [1.1] vs 0.02 [0.2], *P* < .001) and emergency department visits (2.1 [2.6] vs 0.2 [0.7], *P* < .001) per year than low-risk patients during the same period.

**Table 1.** Characteristics and Health Service Use of High-Risk and Low-Risk Veterans^a^

Variable	High-risk veterans (n = 351 012)	Low-risk veterans (n = 3 958 180)	Odds ratio (95% CI) or *P* value^b^
Characteristics, No. (%)			
Male	327 443 (93.3)	3 665 273 (92.6)	1.11 (1.10-1.13)
Not married	218 418 (62.8)	1 639 231 (42.0)	2.33 (2.32-2.35)
Service connection ≥50%^c^	146 181 (72.7)	1 151 896 (55.2)	2.17 (2.14-2.19)
Housing instability	28 805 (8.2)	59 562 (1.5)	5.85 (5.77-5.94)
Age, y			
<45	20 452 (5.8)	631 976 (16.0)	1 [Reference]
45-65	151 613 (43.2)	1 343 279 (33.9)	3.49 (3.44-3.54)
66-75	116 027 (33.1)	1 178 532 (29.8)	3.04 (3.00-3.09)
>75	62 920 (17.9)	804 393 (20.3)	2.42 (2.38-2.46)
Race/ethnicity			
White, non-Hispanic	195 584 (66.2)	2 203 010 (73.8)	1 [Reference]
Black, non-Hispanic	69 554 (23.5)	481 256 (16.1)	1.63 (1.61-1.64)
Hispanic	18 963 (6.4)	188 781 (6.3)	1.13 (1.11-1.15)
Medical comorbidities			
Hypertension	259 361 (73.9)	2 055 111 (52.5)	2.56 (2.54-2.58)
Diabetes	155 226 (44.2)	913 274 (23.1)	2.65 (2.63-2.66)
Asthma or COPD^d^	117 234 (33.4)	425 815 (11.1)	4.03 (4.00-4.06)
Congestive heart failure	82 037 (23.4)	117 827 (3.0)	9.94 (9.85-10.04)
Chronic kidney disease	14 229 (4.1)	22 615 (0.6)	7.35 (7.20-7.51)
Arthritis	178 655 (50.9)	1 300 670 (33.2)	2.08 (2.07-2.10)
Schizophrenia^d^	19 261 (5.5)	43 039 (1.1)	5.14 (5.05-5.22)
Depression^d^	145 738 (41.5)	729 080 (18.6)	3.10 (3.08-3.13)
Alcohol abuse^d^	69 774 (19.9)	202 734 (5.2)	4.54 (4.50-4.59)
Dementia	35 794 (10.2)	101 343 (2.6)	4.32 (4.27-4.38)
Health service use during the past year, mean (SD)			
Any face-to-face primary care encounters^d^	6.3 (6.6)	2.5 (2.9)	<.001
Any primary care telephone encounters^d^	4.0 (4.9)	1.0 (2.1)	<.001
Any primary care secure messages	2.7 (12.7)	1.2 (6.5)	<.001
Hospitalizations^d^	0.8 (1.1)	0.02 (0.2)	<.001
Emergency department visits^d^	2.1 (2.6)	0.2 (0.7)	<.001

Abbreviation: COPD, chronic obstructive pulmonary disease.

^a^ Characteristics or health service use during the year before identification as high or low risk (index date).

^b^ Odds ratios are the odds of being high risk among those having the characteristic compared with the odds of being high risk among those not having the characteristic. The odds ratio can overestimate the relative risk when the outcome of interest occurs in greater than 10% of the sample.

^c^ Service-connected disability refers to a monetary benefit paid to veterans who are determined by the Veterans Health Administration to be disabled by an injury or illness that was incurred or aggravated during active military service.

^d^ An element of the Veterans Health Administration risk score for hospitalization (eTable 1 in the Supplement).

**Figure 1.**
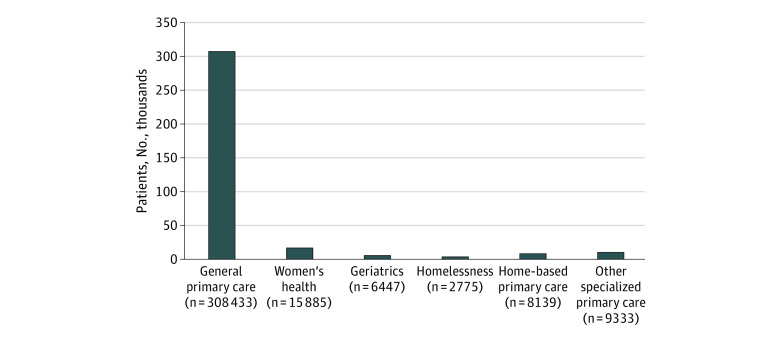
Distribution of Where 351 012 High-Risk Veterans Received Primary Care as of September 30, 2015

Figure 1 shows that most high-risk patients (308 433 of 351 012 [88%]) were assigned to general care than to specialized primary care. Of the remaining high-risk patients, 5% were assigned to women’s health, 2% to geriatrics, 2% to home-based primary care, 0.8% to homelessness primary care, 0.8% to HIV care, 0.8% to spinal cord injury care, 0.2% to dialysis, and 0.9% to all other specialized primary care settings.

**Figure 2.**
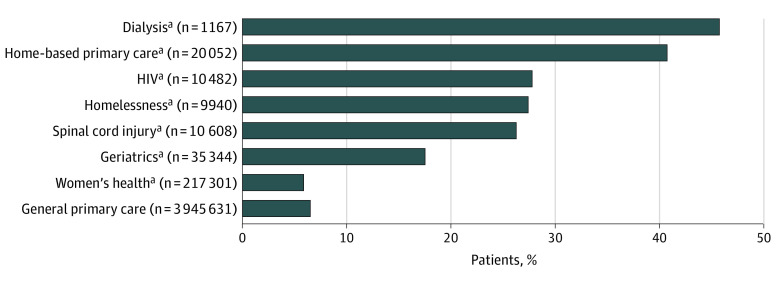
Proportions of 4 309 192 High-Risk Patients in Veterans Health Administration General Primary Care and 7 Specialized Primary Care Settings Data as of September 30, 2015. ^a^*P* < .05 when comparing general primary care with each specialized primary care setting using Tukey-style multiple comparisons of proportions.

As shown in Figure 2, among all patients (both high- and low-risk patients) assigned to general primary care, 8% (308 433 of 3 945 631) were high risk. Among all patients assigned to any specialized primary care setting, 12% (42 579 of 363 561) were high risk. The proportions of high-risk patients varied across the 7 specialized primary care settings, ranging from 46% (532 of 1167) of dialysis primary care to 7% (15 885 of 217 301) of women’s health primary care.

We found varying patterns of primary care assignment when we assessed assignment with respect to potentially qualifying characteristics (age >70 years, homelessness, HIV, and women’s health). Among the 3 more cognitive specialized primary care services, 6% (5821 of 98 181) of high-risk patients older than 70 years were assigned to geriatrics primary care compared with 85% (83 846 of 98 181) in general primary care, with the remaining 9% in other specialized primary care settings. Among high-risk homeless patients, 5% (2468 of 52 506) were assigned to homelessness primary care compared with 85% (44 600 of 52 506) in general primary care, with the remaining 10% in other specialized primary care settings. For high-risk women, 68% (16 020 of 23 569) were assigned to women’s health compared with 28% (6598 of 23 569) assigned to general primary care. For specialized HIV primary care, a highly technical primary care service, 51% (2836 of 5581) of patients diagnosed with HIV infection were assigned to specialized HIV primary care compared with 45% (2533 of 5581) assigned to general primary care.

**Figure 3.**
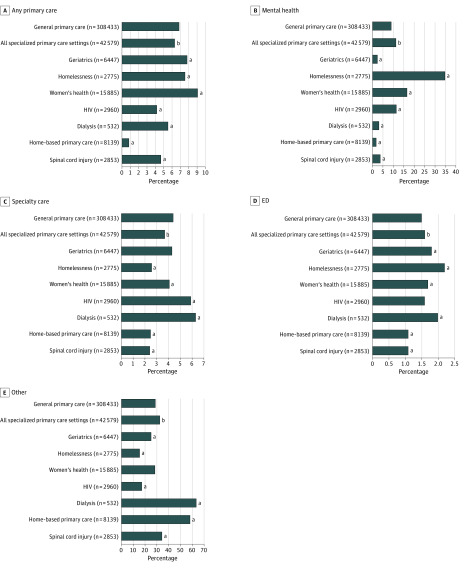
Mean Number of Veterans Health Administration Ambulatory Encounters During 1 Year Among High-Risk Patients by Primary Care Setting Mean counts of any in-person primary care (general or specialized), any mental health, any medical specialty, or any emergency department (ED) face-to-face visit from October 1, 2015, to September 30, 2016. Other encounters include any outpatient telephone, telehealth, surgical, radiology, rehabilitation, and procedural visits. Quantitative results are also given in eTable 2 in the Supplement. Two-sample *t* tests were used for each encounter type comparing patients enrolled in general vs specialized primary care. We performed the Tukey multiple comparison procedure to assess differences in means. ^a^*P* < .05. ^b^*P* < .001.

As shown in Figure 3, general and specialized primary care patients had substantial use of primary care, mental health, and emergency department services during the year after they were identified as high risk (eTable 3 in the Supplement). However, high-risk specialized primary care patients had significantly more outpatient visits (mean [SD], 55.6 [40.3] per year) than did those enrolled in general primary care (mean [SD], 50.5 [35.8] per year; *P* < .001). Compared with specialized primary care patients, general primary care patients had significantly more face-to-face primary care encounters (mean [SD], 6.9 [6.5] per year general vs 6.3 [7.3] per year specialized primary care; *P* < .001) and medical specialty encounters (mean [SD], 4.4 [5.9] per year general vs 3.7 [5.4] per year specialized primary care; *P* < .001). High-risk patients assigned to general primary care had significantly fewer mental health (9.0 [21.6] per year general vs 11.3 [23.9] per year specialized primary care; *P* < .001), emergency department (mean [SD], 1.5 [2.5] per year general vs 1.6 [2.7] per year specialized primary care; *P* < .001), and other (eg, telephone, surgical, telehealth, and physical therapy) (mean [SD], 28.9 [24.5] per year general vs 32.8 [30.8] per year specialized primary care; *P* < .001) encounters. Two types of specialized primary care had substantially higher rates of mental health use than other primary care settings (mean [SD], 16.4 [26.4] per year for women’s health and 34.6 [39.1] per year for homelessness).

Overall, few high-risk patients used add-on services for intensifying primary care (Table 2). Among the 351 012 high-risk patients in our study, 10.9% (38 354) received telehealth monitoring for chronic conditions (eg, hypertension and diabetes), 6.8% (23 827) received housing services, 4.1% (14 545) received palliative care or hospice, and 1.6% (5620) received intensive mental health case management. Different specialized primary care settings, however, used these add-on services at different rates. For example, 4.5% (124 of 2775) of homeless primary care high-risk patients received telehealth services, whereas 53.5% (1484 of 2775) received housing services. In contrast, 15.9% (1291 of 8139) of home-based primary care high-risk patients received telehealth services, whereas 0.7% (70 of 8139) received housing services.

**Table 2.** Receipt of Any Add-on Intensive Services by Primary Care Type From October 1, 2015, Through September 30, 2016

Add-on intensive service	No. (%) of veterans						
	General primary care (n = 308 433)	Specialized primary care					Total (N = 351 012)
		Women’s health (n = 15 885)	Geriatrics (n = 6447)	Homelessness (n = 2775)	Home based (n = 8139)	Other (n = 9333)^a^	
Telehealth services	33 898 (11.0)	1578 10.0)	746 (11.6)	124 (4.5)	1291 (15.9)	717 (7.7)	38 354 (10.9)
Palliative care or hospice services	12 392 (4.0)	302 (1.9)	629 (9.8)	47 (1.7)	859 (10.6)	316 (3.4)	14 545 (4.1)
Intensive mental health case management services	4849 (1.6)	487 (3.1)	33 (0.5)	79 (2.8)	71 (0.9)	101 (1.1)	5620 (1.6)
Housing services	19 927 (6.5)	1525 (9.6)	65 (1.0)	1484 (53.5)	70 (0.9)	756 (8.1)	23 827 (6.8)

^a^ Other refers to specialized primary care for patients with HIV, end-stage renal disease while receiving dialysis, and spinal cord injury.

## Discussion

High-risk patients in the VHA are a small fraction of the general population but account for nearly half of overall health care costs,^40^ most of which are accounted for by hospitalizations.^5^ Contrary to expectations, we found that most high-risk veterans (88%) were assigned to general primary care rather than specialized primary care. Patients assigned to general primary care had more mental health and primary care visits than medical specialty visits. High-risk patients used all types of encounters, including in-person, telephone, and secure messaging, at higher rates than low-risk patients.

Our findings challenge health care systems and researchers to consider the roles, goals, and implications of general primary care for high-risk patient populations. Health care systems are already providing risk-stratified care management, as in the Medicare Comprehensive Primary Care Plus Initiative,^41,42,43^ and turning to specialized programs or intensive primary care models^26^ to manage high-risk patient populations. Although specialized programs or intensive primary care models potentially offer important benefits to high-risk patients, these settings are often more resource intensive than general primary care^26,27,44,45,46,47,48^ and more available in urban areas.^49^

One reason for investing in specialized primary care teams is to provide expertise and resources to special populations that may be more complex than the general primary care population. Overall, we found that most patients assigned to specialized settings were low risk. We also found that even among patients with a technically demanding condition, such as HIV infection, only half of patients with the condition were followed up in specialized primary care. The single exception was for high-risk women veterans, among whom two-thirds were enrolled in specialized women’s health teams. This finding is likely attributable to the need for sex-specific specialized services and procedures^50,51^ and the VHA’s effort to provide specialized support to designated women’s health primary care practitioners at a distance to meet this need, such as through Specialty Care Access Network–Extension for Community Healthcare Outcomes.^52^ Further investigation is warranted.

Our findings suggest that developing population-based approaches for supporting needed high-risk patient care modalities within general primary care settings may be more beneficial than carve-out specialized or intensive primary care programs. Multiple investigations^9,26,41,53,54,55,56,57^ in VHA and non-VHA settings indicate that intensive case management for high-risk patients rarely achieves expected cost savings, whereas access to comprehensive primary care has been associated with improved outcomes, decreased mortality, lower hospitalization rates, emergency department visits, and health care costs both outside^58,59,60,61,62,63^ and inside^22,37,64,65^ the VHA. However, key care modalities known to be needed by high-risk patients are not easily accessible to general primary care teams. For example, time constraints in primary care and training needs can make it challenging to perform assessments for known risk factors, such as falls, cognitive impairment, social isolation, medication adherence, or mental health issues. These assessments may be a starting point for more effective use of specialists and other relevant health care resources for chronic disease management and for care coordination.^32,44,66,67^ In addition, training and support from specialists at a distance^68,69^ or expansion of add-on services could potentially improve general primary care effectiveness for high-risk patients, building on efforts currently under way in the VHA.^70,71^

Our findings also highlight the burden of mental health conditions and high rates of mental health service use among veterans who are at high risk for hospitalization. Further investigations into the patterns of mental health care among high-risk patients may be helpful for developing a focus on the complex needs of these patients within the primary care and mental health integration program.^30^

### Limitations

This study has limitations. It is descriptive and does not address causation. Our VHA-based findings may not be generalizable to other health care systems or to fee-for-service health care settings that may incentivize specialty care.^72^ Our findings have implications, however, for any large system that aims to provide equitable access to care across an enrolled population. Furthermore, although VHA enrollees have higher rates of psychosocial conditions than non-VHA populations,^72,73^ higher psychosocial condition rates are typical for high-risk patients in most health care systems. Additional limitations are that we did not assess non-VA use of care^74^; we studied only how many encounters of particular types occurred without potentially relevant modeling information, such as patient functional status or distance to specialized care settings. Similarly, our data did not support analyses based on specialized primary care enrollment criteria; we used only very general indicators. In addition, our encounter data do not include slight recent VHA coding changes that resulted in a very small proportion of telephone visits being counted as in-person visits; distributions of in-person visits, however, were similar using alternative VHA data definitions.

## Conclusions

Achieving the triple aim of improving patient experience of care, improving population health, and reducing per capita health care costs among high-risk patients^75^ has proved to be elusive.^9,26,41,53,54,55,56^ Our data suggest that a better understanding of existing and optimal roles of different types of primary and specialty care for high-risk patient populations may be critical for achieving the triple aim. Planning for high-risk patient care improvement in integrated health care systems such as the VHA should focus on enabling high-quality complex patient care within primary care and mental health services. In this study, many high-risk veterans were avid users of electronic and telephone care, suggesting that opportunities exist to improve care through maximizing the accessibility and effectiveness of non–face-to-face modalities. Overall, our findings provide a foundation for investigation aimed at informing better design of health care programs and resources that can engage patients in their existing locations of care.

## Notes

Supplement. **eTable 1.** Care Assessment Needs (CAN) 2.0 Model Coefficients, Odds Ratios, and Associated 95% Confidence Intervals for Hospitalization 90-Day Model **eTable 2.** ICD-9 Codes for Selected Medical and Behavioral Conditions **eTable 3.** Average Number of VHA Ambulatory Encounters Over One Year Among High-Risk Patients, by Primary Care Setting^a^ (October 1, 2015 – September 30, 2016) **eTable 4.** Average Number of VHA Ambulatory Encounters Over One Year Among High-Risk Patients Alive Throughout VHA Fiscal Year 2016, by Primary Care Setting^a^ (October 1, 2015 – September 30, 2016) **eTable 5.** Receipt of Any Add-On Intensive Services by Primary Care Type Among High-Risk Patients Alive Throughout VHA Fiscal Year 2016 (October 1, 2015 – September 30, 2016)
