# Supplementary material for: Use of General Primary Care, Specialized Primary Care, and Other Veterans Affairs Services Among High-Risk Veterans
Source: JAMA Netw Open. 2020 Jun 29;3(6):e208120. doi: 10.1001/jamanetworkopen.2020.8120 (PMC7324956; doi:10.1001/jamanetworkopen.2020.8120)
Supplement: Supplement. — eTable 1. Care Assessment Needs (CAN) 2.0 Model Coefficients, Odds Ratios, and Associated 95% Confidence Intervals for Hospitalization 90-Day Model eTable 2. ICD-9 Codes for Selected Medical and Behavioral Conditions eTable 3. Average Number of VHA Ambulatory Encounters Over One Year Among High-Risk Patients, by Primary Care Settinga (October 1, 2015 – September 30, 2016) eTable 4. Average Number of VHA Ambulatory Encounters Over One Year Among High-Risk Patients Alive Throughout VHA Fiscal Year 2016, by Primary Care Settinga (October 1, 2015 – September 30, 2016) eTable 5. Receipt of Any Add-On Intensive Services by Primary Care Type Among High-Risk Patients Alive Throughout VHA Fiscal Year 2016 (October 1, 2015 – September 30, 2016) [file jamanetwopen-3-e208120-s001.pdf]

Supplementary Online Content

Chang ET, Zulman DM, Nelson KM, et al. Use of general primary care, specialized primary care, and other Veterans Affairs services among high-risk veterans. *JAMA Netw Open*. 2020;3(6):e208120. doi:10.1001/jamanetworkopen.2020.8120

eTable 1. Care Assessment Needs (CAN) 2.0 Model Coefficients, Odds Ratios, and Associated 95% Confidence Intervals for Hospitalization 90-Day Model

eTable 2. ICD-9 Codes for Selected Medical and Behavioral Conditions

eTable 3. Average Number of VHA Ambulatory Encounters Over One Year Among High-Risk Patients, by Primary Care Settinga (October 1, 2015 – September 30, 2016)

eTable 4. Average Number of VHA Ambulatory Encounters Over One Year Among High-Risk Patients Alive Throughout VHA Fiscal Year 2016, by Primary Care Setting[a] (October 1, 2015 – September 30, 2016)

eTable 5. Receipt of Any Add-On Intensive Services by Primary Care Type Among High-Risk Patients Alive Throughout VHA Fiscal Year 2016 (October 1, 2015 – September 30, 2016)

This supplementary material has been provided by the authors to give readers additional information about their work.

© 2020 Chang ET et al. *JAMA Network Open*.

eTable 1. Care Assessment Needs (CAN) 2.0 Model Coefficients, Odds Ratios, and Associated 95% Confidence Intervals for Hospitalization 90-Day Model

	**Model Coefficient**			**Odds Ratio**		
	**Hospitalization in 90 days**			**Hospitalization in 90 days**		
	**Estimate**	**95% CI**		**Estimate**	**95% CI**	
**Demographics**						
Age < 55 vs Age >= 85	-0.150	-0.176	-0.124	0.861	0.838	0.883
Age 55-64 vs Age >= 85	-0.047	-0.071	-0.024	0.954	0.932	0.976
Age 65-74 vs Age >= 85	-0.123	-0.145	-0.101	0.884	0.865	0.904
Age 75-84 vs Age >= 85	-0.120	-0.144	-0.097	0.887	0.866	0.908
Not Married vs Married	0.229	0.217	0.242	1.258	1.243	1.273
Vet Priority level 0 vs 5	-0.232	-0.259	-0.204	0.793	0.772	0.815
Vet Priority level 1 vs 5	-0.075	-0.103	-0.046	0.928	0.902	0.955
Vet Priority level 2 vs 5	-0.241	-0.271	-0.212	0.785	0.763	0.809
Vet Priority level 4 vs 5	0.042	0.000	0.085	1.043	1.000	1.088
Enlisted/Unknown vs Officer	0.370	0.311	0.429	1.448	1.365	1.535
SES Index Decile 1 vs Default/No SES Index Calculated	0.015	-0.006	0.036	1.015	0.994	1.037
SES Index Decile 2 vs Default/No SES Index Calculated	-0.039	-0.062	-0.017	0.962	0.940	0.984
SES Index Decile 3 vs Default/No SES Index Calculated	-0.043	-0.067	-0.020	0.957	0.936	0.980
SES Index Decile 4 vs Default/No SES Index Calculated	-0.082	-0.106	-0.058	0.921	0.899	0.943
SES Index Decile 5 vs Default/No SES Index Calculated	-0.107	-0.131	-0.082	0.899	0.877	0.921
SES Index Decile 6 vs Default/No SES Index Calculated	-0.106	-0.131	-0.081	0.900	0.878	0.922
SES Index Decile 7 vs Default/No SES Index Calculated	-0.140	-0.166	-0.115	0.869	0.847	0.891
SES Index Decile 8 vs Default/No SES Index Calculated	-0.119	-0.145	-0.094	0.888	0.865	0.910
SES Index Decile 9 vs Default/No SES Index Calculated	-0.154	-0.180	-0.127	0.858	0.835	0.880
SES Index Decile 10 vs Default/No SES Index Calculated	-0.181	-0.208	-0.154	0.835	0.812	0.858
**Vital Signs**						
Body Mass Index: 0/Unknown vs > 40	0.420	0.353	0.487	1.522	1.423	1.627
Body Mass Index: 16 <= BMI <= 40 vs > 40	0.040	0.016	0.064	1.040	1.016	1.066
Coefficient of Variation for Weight: <= 4 vs > 4	-0.133	-0.145	-0.120	0.876	0.865	0.887
Most Recent Pulse Vital Measurement prior 1 year: < 60 vs >= 90	-0.286	-0.309	-0.263	0.751	0.734	0.769
Most Recent Pulse Vital Measurement prior 1 year: 60 <= pulse < 90 or Unknown vs >= 90	-0.212	-0.227	-0.197	0.809	0.797	0.821

© 2020 Chang ET et al. *JAMA Network Open*.

Most Recent Systolic Blood Pressure Measurement prior 1 year: < 110 vs >= 160	-0.152	-0.181	-0.123	0.859	0.834	0.884
Most Recent Systolic Blood Pressure Measurement prior 1 year: 110 <= syst < 140 or Unknown vs >= 160	-0.255	-0.279	-0.231	0.775	0.756	0.794
Most Recent Systolic Blood Pressure Measurement prior 1 year: 140 <= syst < 160 vs >= 160	-0.153	-0.180	-0.127	0.858	0.835	0.881
**Medical and Psychiatric Comorbidities**						
Charlson-Deyo Mortality Score: 0 vs > 4	-0.390	-0.413	-0.367	0.677	0.661	0.693
Charlson-Deyo Mortality Score: 1 vs > 4	-0.311	-0.333	-0.289	0.733	0.717	0.749
Charlson-Deyo Mortality Score: 2 vs > 4	-0.190	-0.213	-0.167	0.827	0.809	0.846
Charlson-Deyo Mortality Score: 3-4 vs > 4	-0.137	-0.158	-0.117	0.872	0.854	0.890
No Mental Disorder Dx & No PTSD Dx vs Mental Disorder Dx or PTSD Dx prior 1 year	-0.111	-0.126	-0.097	0.895	0.882	0.908
Mental Disorder Dx & PTSD Dx vs Mental Disorder Dx or PTSD Dx prior 1 year	-0.065	-0.082	-0.047	0.937	0.921	0.954
Alcohol Dxs prior 2 years: 0-1 vs > 1	-0.281	-0.299	-0.263	0.755	0.742	0.768
Chronic Airway Obstruction Dxs prior 2 years: 0 vs > 0	-0.144	-0.159	-0.129	0.866	0.853	0.879
No Dementia Dx vs Dementia Dx prior 1 year	-0.143	-0.166	-0.121	0.867	0.847	0.886
**Medications**						
No Antipsychotic Medication Filled prior 1 year vs Antipsychotic Med Filled	-0.167	-0.186	-0.148	0.846	0.830	0.862
No BetaBlocker Medication Filled prior 1 year vs BetaBlocker Med Filled	-0.171	-0.185	-0.158	0.842	0.831	0.854
No Furosemide Medication Filled prior 1 year vs Furosemide Med Filled	-0.203	-0.220	-0.186	0.816	0.802	0.830
No Hydroxymethylglutarly-CoenzymeA Medication Filled prior 1 year vs HMGCOA Med Filled	0.092	0.079	0.105	1.097	1.083	1.111
**Laboratory and Radiology Measures**						
Most Recent Albumin Lab result prior 1 year: > 3.4 vs No Albumin Lab	-0.110	-0.126	-0.094	0.896	0.882	0.910
Most Recent Albumin Lab result prior 1 year: 0-3.4 vs No Albumin Lab	0.137	0.114	0.160	1.147	1.121	1.173
Low Red Blood Count Labs prior 2 years: 0 vs > 9	-0.216	-0.239	-0.193	0.806	0.787	0.825
Low Red Blood Count Labs prior 2 years: 1-9 vs > 9	-0.077	-0.098	-0.055	0.926	0.906	0.946
Low Sodium Labs prior 2 years: 0 vs > 9	-0.095	-0.130	-0.060	0.909	0.878	0.941
Low Sodium Labs prior 2 years: 1 vs > 9	-0.007	-0.044	0.030	0.993	0.957	1.030
Low Sodium Labs prior 2 years: 2-4 vs > 9	-0.002	-0.039	0.034	0.998	0.962	1.035
Low Sodium Labs prior 2 years: 5-9 vs > 9	0.018	-0.023	0.058	1.018	0.978	1.060
High White Blood Cell Labs prior 2 years: 0 vs > 1	-0.146	-0.162	-0.131	0.864	0.851	0.877
High White Blood Cell Labs prior 2 years: 1 vs > 1	-0.013	-0.035	0.008	0.987	0.966	1.008
Chest Xray CPTs prior 90 days: 0 vs > 2	-0.468	-0.510	-0.426	0.626	0.601	0.653

© 2020 Chang ET et al. *JAMA Network Open*.

Chest Xray CPTs prior 90 days: 1-2 vs > 2	-0.047	-0.089	-0.004	0.954	0.915	0.996
**Inpatient and Outpatient Utilization Measures**						
Hosp Stays & Bed Days of Care prior 1 year: level 0 vs 4	0.689	0.638	0.740	1.991	1.892	2.096
Hosp Stays & Bed Days of Care prior 1 year: level 1 vs 4	0.292	0.243	0.341	1.339	1.275	1.407
Hosp Stays & Bed Days of Care prior 1 year: level 2 vs 4	0.064	-0.004	0.132	1.066	0.996	1.141
Hosp Stays & Bed Days of Care prior 1 year: level 3 vs 4	0.117	0.080	0.154	1.124	1.083	1.167
All Hospital Admissions prior 1 year: 0 vs > 2	-1.238	-1.283	-1.192	0.290	0.277	0.304
All Hospital Admissions prior 1 year: 1 vs > 2	-0.613	-0.656	-0.570	0.542	0.519	0.566
All Hospital Admissions prior 1 year: 2 vs > 2	-0.360	-0.392	-0.328	0.698	0.676	0.720
Emergency Room Stop Code Visits prior 1 year: 0 vs > 2	-0.479	-0.498	-0.460	0.619	0.608	0.631
Emergency Room Stop Code Visits prior 1 year: 1-2 vs > 2	-0.124	-0.142	-0.106	0.883	0.868	0.899
Established Office Visit CPTs prior 90 days: 0 vs > 4	-0.434	-0.455	-0.412	0.648	0.635	0.662
Established Office Visit CPTs prior 90 days: 1-4 vs > 4	-0.210	-0.227	-0.193	0.810	0.797	0.824
Phone CPTs 21to30 mins prior 2 years: 0 vs > 1	-0.173	-0.190	-0.155	0.841	0.827	0.856
Phone CPTs 21to30 mins prior 2 years: 1 vs > 1	-0.053	-0.074	-0.032	0.949	0.929	0.968
Other Non-Face Stop Code Visits prior 1 year: 0 vs > 15	-1.121	-1.172	-1.070	0.326	0.310	0.343
Other Non-Face Stop Code Visits prior 1 year: 1-2 vs > 15	-0.704	-0.737	-0.671	0.495	0.478	0.511
Other Non-Face Stop Code Visits prior 1 year: 3-4 vs > 15	-0.464	-0.492	-0.435	0.629	0.611	0.647
Other Non-Face Stop Code Visits prior 1 year: 5-9 vs > 15	-0.196	-0.217	-0.176	0.822	0.805	0.839
Other Non-Face Stop Code Visits prior 1 year: 10-15 vs > 15	-0.029	-0.046	-0.011	0.972	0.955	0.989
Primary Care Stop Code Visits prior 1 year: 0 vs > 6	0.252	0.218	0.286	1.286	1.243	1.330
Primary Care Stop Code Visits prior 1 year: 1 vs > 6	-0.051	-0.075	-0.026	0.951	0.927	0.974
Primary Care Stop Code Visits prior 1 year: 2 vs > 6	-0.044	-0.065	-0.023	0.957	0.937	0.977
Primary Care Stop Code Visits prior 1 year: 3-4 vs > 6	-0.007	-0.024	0.009	0.993	0.977	1.009
Primary Care Stop Code Visits prior 1 year: 5-6 vs > 6	0.000	-0.017	0.018	1.000	0.983	1.018
Phone Stop Code Visits prior 1 year: 0 vs > 3	-0.096	-0.118	-0.073	0.909	0.889	0.929
Phone Stop Code Visits prior 1 year: 1-3 vs > 3	-0.038	-0.062	-0.013	0.963	0.940	0.987
CT Primary Stop Code Visits prior 1 year: 0 vs > 3	-0.256	-0.279	-0.233	0.774	0.757	0.792
CT Primary Stop Code Visits prior 1 year: 1-3 vs > 3	-0.055	-0.078	-0.032	0.947	0.925	0.968
Cardiology Stop Code Visits prior 1 month: 0 vs > 0	-0.391	-0.420	-0.363	0.676	0.657	0.696
TIU Consent Notes prior 1 year: 0 vs > 4	-0.146	-0.172	-0.119	0.865	0.842	0.888
TIU Consent Notes prior 1 year: 1 vs > 4	-0.037	-0.064	-0.010	0.964	0.938	0.990
TIU Consent Notes prior 1 year: 2-4 vs > 4	-0.022	-0.048	0.003	0.978	0.953	1.003
TIU Telephone Notes prior 2 years: 0 vs > 9	-0.218	-0.243	-0.193	0.804	0.784	0.825
TIU Telephone Notes prior 2 years: 1-4 vs > 9	-0.038	-0.054	-0.021	0.963	0.947	0.979
TIU Telephone Notes prior 2 years: 5-9 vs > 9	0.014	-0.002	0.030	1.014	0.998	1.031
Outpatient Visits prior 3 years: 0-90 vs > 180	-0.494	-0.516	-0.472	0.610	0.597	0.623

© 2020 Chang ET et al. *JAMA Network Open*.

Outpatient Visits prior 3 years: 91-180 vs > 180	-0.172	-0.188	-0.155	0.842	0.828	0.857
Intercept	-1.399					

Model Calibration	AUC
90 – Day Event	0.81
1 – Year Event	0.79
90 – Day Hospitalization	0.83
1 – Year Hospitalization	0.81
90 – Day Mortality	0.87
1 – Year Mortality	0.86

© 2020 Chang ET et al. *JAMA Network Open*.

eTable 2. ICD-9 Codes for Selected Medical and Behavioral Conditions

ICD-9 Description	ICD-9 CM Code
Hypertension	401.0, 401.1, 401.9, 402.10, 402.90, 402, 404.10, 404.90, 404, 405.11, 405.19, 405.91, 405.99
Diabetes	250.xx
Depression	293.83, 296.2, 296.3, 296.82, 296.90, 296.99, 300.4, 311
Asthma or COPD	493, 494, 496, 491.0, 491.1, 491.2, 491.8, 491.9, 492.0, 492.8
Congestive Heart Failure	402.01, 402.11, 402.91, 404.01, 404.03, 404.11,404.13,404.91, 404.93, 428, 425
Arthritis	710.0, 710.1, 712, 713, 714, 715, 719.3, 720, 721,716.5-716.9, 719.4-719.6, 719.8, 719.9
Chronic Kidney Disease	403.01, 403.11, 403.91, 404.02, 404.03, 404.12, 404.13, 404.92, 404.93, 585.5, 585.6 all stage V or End Stage, all V45.1 and V56 dialysis, E879.1
Alcohol Abuse	303.01, 303.90, 303.91, 303.92, 305.00, 305.01, 305.02
Homeless	V60.0
Schizophrenia	295.xx
Human Immunodeficiency Virus (HIV)	042.xx
Dementia	331.0-331.2, 290.xx, 291.2, 292.82, 294.1x, 294.2x, 331.82

© 2020 Chang ET et al. *JAMA Network Open*.

eTable 3. Average Number of VHA Ambulatory Encounters Over One Year Among High-Risk Patients, by Primary Care Setting[a] (October 1, 2015 – September 30, 2016)

	N	Visits in one year, Mean (SD)	Visits to Any Primary Care, Mean (SD)	Visits to Mental Health, Mean (SD)	Visits to Specialty Care, Mean (SD)	Visits to Emergency Room, Mean (SD)	Other visits, Mean (SD)
General Primary Care	308,433	50.5 (35.8)	6.9 (6.5)	9.0 (21.6)	4.4 (5.9)	1.5 (2.5)	28.9 (24.5)
**All Specialized Primary Care**	42,579	55.6 (40.3)**	6.3 (7.3)**	11.3 (23.9)**	3.7 (5.4)**	1.6 (2.7)**	32.8 (30.8)**
• Women's Health	15,885	59.9 (38.4)*	9.1 (8.1)*	16.4 (26.4)*	4.1 (5.7)*	1.7 (2.6)*	28.7 (22.5)
• Geriatrics	6,447	41.0 (31.0)*	7.8 (6.6)*	2.3 (9.2)*	4.3 (5.5)	1.8 (2.6)*	24.8 (23.3)*
• Homeless	2,775	61.5 (48.2)*	7.6 (8.1)*	34.6 (39.1)*	2.6 (4.0)*	2.2 (4.2)*	15.3 (18.8)*
• Home-based Primary Care	8,139	64.7 (42.0)*	0.9 (2.9)*	1.9 (8.2)*	2.5 (4.0)*	1.1 (2.0)*	58.3 (38.0)*
• Human Immunodeficiency Virus	2,960	40.5 (38.0)*	4.2 (4.5)*	11.6 (23.1)*	5.9 (7.1)*	1.6 (2.7)	17.3 (22.1)*
• Spinal Cord Injury	2,853	46.2 (36.0)*	4.7 (6.6)*	3.6 (11.9)*	2.4 (4.0)*	1.1 (2.1)*	34.4 (29.1)*
• Dialysis	532	80.7 (68.1)*	5.5 (6.2)*	3.2 (10.7)*	6.3 (8.6)*	2.0 (2.8)*	63.8 (64.5)*

[a]All encounters are face-to-face, except other includes face-to-face, telephone, and virtual encounters.
Comparisons made using general primary care as reference for each visit type. Tukey's multiple comparison procedure used to account for multiple comparisons **p<0.001; *p<0.05.

© 2020 Chang ET et al. *JAMA Network Open*.

eTable 4. Average Number of VHA Ambulatory Encounters Over One Year Among High-Risk Patients Alive Throughout VHA Fiscal Year 2016, by Primary Care Setting[a] (October 1, 2015 – September 30, 2016)

	N	Visits in one year, Mean (SD)	Visits to Any Primary Care, Mean (SD)	Visits to Mental Health, Mean (SD)	Visits to Specialty Care, Mean (SD)	Visits to Emergency Room, Mean (SD)	Other visits, Mean (SD)
General Primary Care	306,639	50.8 (35.7)	6.9 (6.5)	9.0 (21.7)	4.4 (5.9)	1.5 (2.5)	29.0 (24.5)
**All Specialized Primary Care**	42,285	56.0 (40.2)**	6.4 (7.3)**	11.4 (23.9)**	3.7 (5.4)**	1.6 (2.7)**	33.0 (30.8)**
• Women's Health	15,851	60.0 (38.4)*	9.1 (8.0)*	16.4 (26.4)*	4.1 (5.7)*	1.7 (2.7)*	28.8 (22.5)
• Geriatrics	6,386	41.4 (30.9)*	7.8 (6.6)*	2.4 (9.3)*	4.3 (5.5)	1.8 (2.6)*	25.1 (23.3)*
• Homeless	2,746	62.1 (48.1)*	7.7 (8.1)*	34.9 (39.1)*	2.6 (4.0)*	2.2 (4.2)*	15.4 (18.9)*
• Home-based Primary Care	8,016	65.7 (41.5)*	0.9 (2.9)*	1.9 (8.2)*	2.6 (4.0)*	1.1 (2.0)*	59.2 (37.6)*
• Human Immunodeficiency Virus	2,955	40.5 (38.0)*	4.2 (4.5)*	11.7 (23.1)*	5.9 (7.2)*	1.6 (2.7)	17.3 (22.1)*
• Spinal Cord Injury	2,823	46.7 (36.0)*	4.8 (6.6)*	3.6 (12.0)*	2.4 (4.1)*	1.1 (2.1)*	34.8 (29.1)*
• Dialysis	528	81.2 (68.1)*	5.5 (6.2)*	3.2 (10.7)*	6.4 (8.6)*	2.0 (2.8)*	64.2 (64.6)*

[a]All encounters are face-to-face, except other includes face-to-face, telephone, and virtual encounters.
Comparisons made using general primary care as reference for each visit type. Tukey's multiple comparison procedure used to account for multiple comparisons **p<0.001; *p<0.05.

© 2020 Chang ET et al. *JAMA Network Open*.

eTable 5. Receipt of Any Add-On Intensive Services by Primary Care Type Among High-Risk Patients Alive Throughout VHA Fiscal Year 2016 (October 1, 2015 – September 30, 2016)

Add-on Intensive Service	General Primary Care (n=306,639)		Specialized Primary Care: Women's Health (n=15,851)		Geriatrics (n=6,386)		Homeless (n=2,746)		Home-Based (n=8,016)		Other[a] (n=9,286)		Total (n=348,924)	
	N	(%)	N	(%)	N	(%)	N	(%)	N	(%)	N	(%)	N	(%)
**Telehealth Services**	33,784	(11.0)	1,571	(9.9)	744	(11.7)	123	(4.5)	1,283	(16.0)	713	(7.7)	38,218	(11.0)
**Palliative care or hospice services**	11,931	(3.9)	295	(1.9)	613	(9.6)	42 (1.5)		827	(10.3)	301	(3.2)	14,009	(4.0)
**Intensive mental health case management services**	4,840	(1.6)	485	(3.1)	33 (0.5)		78 (2.8)		71	(0.9)	101	(1.1)	5,608	(1.6)
**Housing services**	19,870	(6.5)	1,524	(9.6)	65 (1.0)		1,472	(53.6)	69	(0.9)	755	(8.13)	23,755	(6.8)

[a] "Other" refers to specialized primary care for patients with HIV, end-stage renal disease on dialysis, and spinal cord injury.

© 2020 Chang ET et al. *JAMA Network Open*.
